# Effects of Physical Activity and Nutrition Education on the Gut Microbiota in Overweight and Obese Children

**DOI:** 10.3390/children10071242

**Published:** 2023-07-19

**Authors:** Micaela C. Morgado, Mónica Sousa, Cláudia Marques, André B. Coelho, Júlio A. Costa, André Seabra

**Affiliations:** 1 Research Centre in Physical Activity, Health and Leisure (CIAFEL), Faculty of Sport, University of Porto, 4200-450 Porto, Portugal; mmorgado.nutritionist@outlook.com; 2 Portugal Football School, Portuguese Football Federation (FPF), 1495-433 Cruz Quebrada, Portugal; jahdc@hotmail.com; 3 CINTESIS@RISE, NOVA Medical School (NMS), Faculdade de Ciências Médicas (FCM), Universidade Nova de Lisboa, 1169-056 Lisboa, Portugal; monicavcsousa@gmail.com (M.S.); claudia.sofia.marques@nms.unl.pt (C.M.); 4 Faculty of Sports Science and Physical Education, University of Coimbra, 3040-248 Coimbra, Portugal; andrebastoscoelho@hotmail.com

**Keywords:** childhood obesity, gut microbiota, physical activity, nutrition, football, *Bifidobacterium*, *Prevotella*, Firmicutes-to-Bacteroidetes ratio

## Abstract

Childhood obesity continues to represent a growing challenge, and it has been associated with gut microbiota dysbiosis. This study examines the gut microbiota composition in overweight and obese school children and assesses whether a 12-week multidisciplinary intervention can induce changes in the gut microbiota. The intervention, which combined recreational football and nutritional education, was implemented among 15 school children, aged 7–10 years, with a Body Mass Index ≥ 85th percentile. The children were assigned into two groups: Football Group (*n* = 9) and Nutrition and Football Group (*n* = 6). Faecal samples were collected at the beginning and end of the program and analysed by sequencing the 16S rRNA gene. Over the intervention, a significant decrease was found collectively for Bifidobacterium genera (*p* = 0.011) and for *Roseburia* genera in the Football Group (*p* = 0.021). The relative abundance of *Roseburia* (*p* = 0.002) and *Roseburia faecis* (*p* = 0.009) was negatively correlated with moderate to vigorous physical activity (MVPA), while *Prevotella copri* was positively correlated with MVPA (*p* = 0.010) and with the daily intake of protein (*p* = 0.008). Our findings suggest that a multidisciplinary intervention was capable of inducing limited but significant positive changes in the gut microbiota composition in overweight and obese school children.

## 1. Introduction

Overweight and obesity affect nearly one in three children in the WHO European Region, being a target of policy options to prevent obesity worldwide [1]. Obesity is a complex, multifactorial condition affected by numerous social, environmental, and economic exposures that interact with individual behavioural and biological factors to accumulate across the course of life [2]. Family factors, including the relational dynamics during meals in mother–child pairs [3] and maternal malnutrition during the child-rearing period [4], could potentially influence the progress of children’s body weight. Children with obesity are more prone to become adults affected by obesity and its multiple serious obesity-related comorbidities [5], particularly those with severe obesity and/or a strong family history of obesity [6,7,8,9].

Overweight and obesity comes as a result of energy imbalance between energy intake and expenditure over a period of time [10]. While higher energy intake and less physical activity among children contribute to overweight and obesity, there is also growing awareness of variation in the gut microbiota of children with obesity compared to those of normal weight [11]. Recent literature has revealed that obesity in children is associated with gut microbial dysbiosis [12,13]. In addition, variations in gut microbiome composition and relative diversity have been found to be associated with weight status in early childhood [14]. Obesity seems to be associated with changes in the Firmicutes/Bacteroidetes ratio (F/B ratio), especially for a reduction in the relative abundance of Bacteroidetes [15]. An elevated F/B ratio has been shown in children with obesity when compared with lean counterparts [11,12].

In the case of obese children, studies suggest that physical activity is the best tool to reduce abdominal obesity and cardiovascular risk and improve metabolic parameters [16]. Physical activity is also one of the factors that seems to influence the gut microbiota and that can have a positive impact on health with increased biodiversity and the presence of taxa with beneficial metabolic functions [17]. In physical activity interventions with overweight and obese children it was suggested that daily exercise increases gut microbial diversity with a Firmicutes phylum enrichment [18,19,20]. However, research on microbial changes in overweight children after physical activity, weight reduction and nutritional interventions has remained scarce [21,22].

Diet choices have also been suggested as a major driver of the composition and diversity of the gut microbiota [23,24,25]. Several studies have been conducted in infancy and toddlers, but there appears to be a gap in older children regarding the influence of nutrition on gut microbiota composition. It is necessary to study older children to help identify opportunities for intervention [23].

Childhood is a critical stage in the development of the microbiota due to the great plasticity of the gut ecosystem in this period [18]. Furthermore, it has been suggested that the microbiota continues to develop through childhood and children may be the best candidates for microbiota interventions to realize health promotion or disease prevention [21]. Thus, we hypothesized that a combined program with 12-week of recreational football and nutritional education would be capable of inducing changes in the gut microbiota composition, of modifying the Firmicutes to Bacteroidetes ratio, and of having positive effects in body composition and eating habits in overweight and obese elementary school children.

## 2. Materials and Methods

### 2.1. Study Design and Participants

This study is a nonrandomized controlled clinical trial, conducted in elementary schools in central Portugal, approved by the Ethical Committee of the Faculty of Sport of the University of Porto (nr. CEFADE 05 2019). A total of 17 overweight and obese Portuguese elementary school children, with a body mass index (BMI) ≥ 85th percentile [26], aged 7–10 years, agreed to participate after written informed consent was provided by their legal guardians. From these seventeen, two were excluded from the analysis. The reasons for exclusion were as follows: only one faecal sample was collected (*n* = 1); and low training attendance (*n* = 1). A total of 15 children consisting of 47% boys and 53% girls, completed both pre- and post-intervention tests. Among them, 33% were classified as overweight, while the remaining 67% were categorized as obese children. Children were divided into two groups: Football Group (FG, *n* = 9, school I) or Nutrition and Football Group (NFG, *n* = 6, school II). The intervention period lasted from October 2019 to March 2020, and the duration of the intervention was 14 weeks: In week 1, children completed the preintervention testing (anthropometric measurements and body composition, physical activity, dietary assessment and faecal sample collection); in weeks 2–13, children performed the “Football and Nutrition for Health” program: FG performed 2 sessions per week of 60 min of recreational football, and NFG performed 2 sessions per week of 60 min of recreational football plus 60 min of nutritional education; in week 14, post-intervention testing with the same test battery was carried out. The use of medication or the presence of another pathology or clinical condition in which physical activity is contraindicated was consider an exclusion factor, including antibiotic treatment within the previous 3 months and the presence of gastrointestinal comorbidities or cardiovascular disease from the time of faecal sample collection [27]. The participants were also required to take no antibiotics, probiotics, or other supplements during the intervention.

### 2.2. “Football and Nutrition for Health” Intervention Program

**Figure 1:**
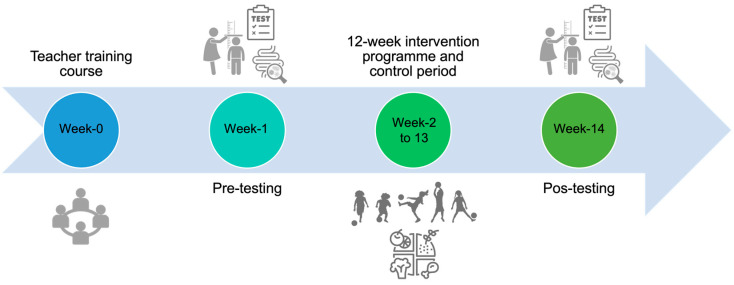
Timeline of the “Football and Nutrition for Health” intervention.

In the “Football and Nutrition for Health” study, physical activity was incorporated based on recreational football practice and nutrition education in the school curriculum (Figure 1). The recreational football sessions were based on the structure, content, and implementation protocol for the “FIFA 11 for Health” program [28,29] with adaptation to 12 weeks, with 2 sessions of 60 min per week, each consisting of a Play Football period (teaching specific football skills and recreational small-sided football games). The nutrition education program consisted in a combination of six health and nutrition issues, on a weekly basis, 60 min for 12 weeks, based on the principles of the Portuguese food wheel: the Portuguese food wheel rules and groups [30]; what are calories, macro and micronutrients; the nutritional traffic light label; how to prepare healthy meals; importance of fruits and vegetables; proof of the sea: choose fish, choose health.

### 2.3. Anthropometric Measurements and Body Composition

Weight, height and waist circumference were collected according to the International Society for the Advancement of Kinanthropometry (ISAK) protocol [31] and BMI (kg·m^−2^) was calculated. Height was measured with a mobile stadiometer (Seca 213, Hamburg, Germany) and perimeters with a metallic tape (Holtain Ltd., Crymych, UK). The participants were weighed bare foot in light clothes, between 9:00 and 10:00, after having breakfast before 8:00. To define children’s nutritional status, z-scores or percentiles of BMI for age and sex according to the criteria and cut-offs defined by the World Health Organization [32,33] and to the growth charts of the Centers for Disease Control and Prevention [26] were considered. Children were classified as overweight if their age- and sex-specific BMI was equal or higher than 85th percentile, and obese if their age- and sex-specific BMI was in the 95th percentile or greater [26]. Waist circumference was measured at the superior border of the iliac crest, according to the protocol of the National Health and Nutrition Examination Survey (NHANES) [34]. To study the central fatness, we used waist-to-height ratios (WtHr). WtHr was calculated by dividing waist circumference (in cm) by height (in cm). Body composition was measured using the classic unifrequency electrical bioimpedance method (Akern body composition analyser, Model BIA101) to estimate body weight, percentage of body fat mass (%BFM), fat-free mass (FFM) and muscle mass (MM), according to protocol [35]. Participants were asked to not practice physical activity in the previous 24 h, to be preferably fasted or at least 4 h without eating and drinking (but not dehydrated), not to ingest diuretics (tea or coffee), to empty their bladder and bowel and, during the test, to remove all metal (bracelets, earrings, etc.) [36].

### 2.4. Physical Activity

To estimate daily physical activity a tri-axial accelerometer (ActiGraph, model GT3X, Acticorp Co., Pensacola, FL, USA) was used at baseline and at the conclusion of the study. ActiLife Software v6.13.4 was used for data processing. Before each testing session, the ActiGraph was initialized according to the manufacturer’s specifications [37]. The ActiGraph was attached to a flexible elastic belt that was fastened snugly around the waist of each child, to remain tight but not too tight. We advised that it is not visible so that other children were not tempted to touch [37]. Children were asked to wear the accelerometer as soon as they got up in the morning (on waking up) and taken out at night to sleep. We also ask to only remove it for sleeping, bathing, during water-based activities and in exceptional cases like while performing contact sports such as martial arts, because of the risk of injury. Accelerometer data files were collected in 15 s epochs according to the cut point chosen to record the spontaneous and intermittent activities of children more accurately [38,39]. The accelerometers were used for 7 consecutive days and the records of physical activity performed in at least 4 days were considered valid [39]. Wear time validation was calculated using Troiano defaults [40,41], and we considered days with ≥480 min of activity recordings as valid [42]. Non-wear time was defined as 60 min of consecutive zeros allowing for 2 min of non-zero interruptions [43,44]. Average counts per minute (CPM) were used as a measure of total physical activity. Evenson cut-points [38], validated cut-points recommended for children, were used to estimate time spent in sedentary, light, moderate, and vigorous intensity activity in children: light (101 to ≥2295), moderate (≥2296 CPM) and vigorous intensity (≥4012 CPM) physical activity [37,39,42,43]. The numbers of minutes per day at different intensities were determined by summing all minutes where the activity count was equal to and greater than the threshold for that intensity, divided by the number of valid days [42].

### 2.5. Dietary Assessment

The collection of dietary intakes was evaluated by a 24 h recall, at baseline and after the intervention, using portion quantification methods with photography of home measurements (cups, bowls and glasses) completed by the legal representatives [45]. Detailed instructions were given to legal representatives to record all foods and beverages consumed by the child, to represent the usual consumption. The instructions consist of discriminating the foods consumed, reporting the commercial name (if applicable) and the portion consumed (in weight, volume, or household measures). In the case of prepared dishes, an indication is given to provide details of the recipe, including ingredients and cooking methods. Information such as mealtime, name of meal, location of meal and day of the week were also reported. At the end of each food record, the form asked if the registered day represents a day of usual consumption and if not, the reasons are asked. There is also an open section for comments. For nutritional data analysis, the ESHA’s Food Processor Nutrition Analysis software, version 11.5, was used. The use of nutritional supplements was assessed through questions of propensity for habitual consumption, from a pre-defined list of different supplements, currently in use in the market, with the possibility of mentioning others not included in the initial list [45]. The reference period to which the use of supplements refers was based on the last month.

### 2.6. Faecal Sample Collection and DNA Extraction

Children and their legal representatives were asked to collect their own faecal samples using an appropriate collection kit (EasySampler^®^, ALPCO, Salem, NH, USA) containing RNAlater (Sigma-Aldrich, St. Louis, MO, USA). The faecal samples were kept at −20 °C until DNA extraction. Faecal samples were collected at two moments: at the beginning (week-1) and end of the intervention (week-14). Bacterial DNA was extracted and purified from all faecal samples using a NZY Tissue gDNA Isolation Kit (NZYtech, Lisbon, Portugal) [46].

### 2.7. Microbial 16S rRNA Sequence Analysis

All 16S DNA libraries (V4 regions) were processed and sequenced following the 16S Metagenomic Sequencing Library Preparation protocol from illumina (illumina; San Diego, CA, USA). Primers used to capture the region V4 of the bacterial 16S region (primers 341F:5′-CCTACGGGNGGCW GCAG-3′, 806R: 5′-GGACTACHVGGGTATCTAAT-3′) [47]. The samples were pooled and loaded into the illumina MiSeq System and sequenced using a 280-multiplex approach on a 2 × 250 bp run, according to manufacturer’s procedures [48].

### 2.8. Bioinformatic Analysis

The microbiome data analysis was performed with QIIME 2 v2020.11 platform [49]. The percentage of features to remove based on low variance was set to 10%, using the interquartile range. To explore the samples taxonomic composition, the assignation was applied by using a pre-trained Naïve Bayes classifier on the GreenGenes (version 13_8) database where OTUs have been generated with a collapsing threshold of 99% sequence similarity. Alpha and beta diversity core metrics were computed through q2-diversity plug-in by rarefying the samples at 19,000 counts sampling depth. Alpha diversity was measured by Shannon’s diversity index that summarizes the species richness and evenness within a sample. The beta-diversity was based on Bray–Curtis distances to evaluate differences in the community of bacterial species over the time factor. Bray–Curtis distances were evaluated between the different experimental groups through permutational multivariate analysis of variance (PERMANOVA) test.

### 2.9. Statistical Analysis

Descriptive statistics (means and standard deviations) were calculated for the groups at baseline and after the intervention. The Shapiro–Wilk test was used to assess the normality of the data. Differences in variables between groups at baseline and after the intervention were determined by applying the Mann–Whitney U-test. The Wilcoxon signed-rank test was used to analyse the effect of the football and nutritional intervention program from baseline to post-intervention. Percentage change (%Δ) between baseline and post-intervention were calculated for each variable. Spearman’s rank correlation was used to assess the relationship between the relative abundance of the most abundant bacteria and body composition, physical activity, and diet. The absolute magnitude of the correlation coefficient was interpreted as negligible (0.00–0.10), weak (0.10–0.39), moderate (0.40–0.69), strong (0.70–0.89) or very strong (0.90–1.00) [50]. Significance level was set at 0.05. Statistical analyses were conducted using SPSS version 27.0.

## 3. Results

### 3.1. Anthropometric, Body Composition, and Physical Activity

The mean ages of the overweight and obese children were 8.8 ± 0.8 years in the FG and 9.4 ± 0.2 years in the NFG with no differences between groups (*p* > 0.05). No significant differences between participants in anthropometric, body composition, and physical activity were found at baseline (*p* > 0.05).

From baseline to post intervention, both groups showed significant decreases in BMI (FG: Z = −2.314; *p* = 0.021; NFG: Z = −2.201; *p* = 0.028), BMI z-score (FG: Z = −2.556; *p* = 0.011; NFG: Z = −2.201; *p* = 0.028) and WtHr (FG: Z = −1.960; *p* = 0.050; NFG: Z = −2.201; *p* = 0.028), with no differences between groups (*p* > 0.05). Furthermore, the FG showed significant decreases in %BFM (Z = −2.556; *p* = 0.11) and improvements in FFM (Z = −2.668; *p* = 0.008), MM (Z = −2.524; *p* = 0.012) and moderate to vigorous physical activity (MVPA) (Z = −2.429; *p* = 0.015). While the NFG revealed significant differences in the intervention in the reduction in waist circumference (Z = −2.201; *p* = 0.028). No significant differences in body weight over the 12-week intervention was found for either group (*p* > 0.05). Participant characteristics are presented in Table 1.

### 3.2. Gut Microbiota Profile of Overweight and Obese School Children

**Figure 2:**
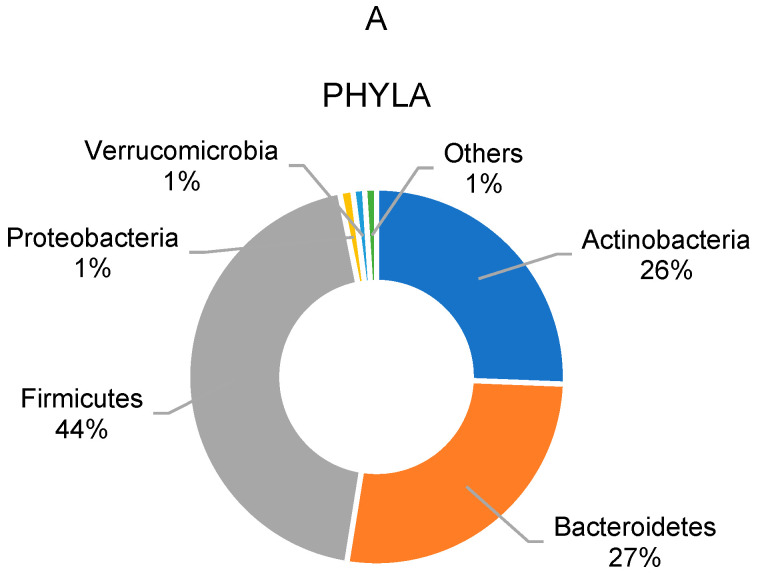

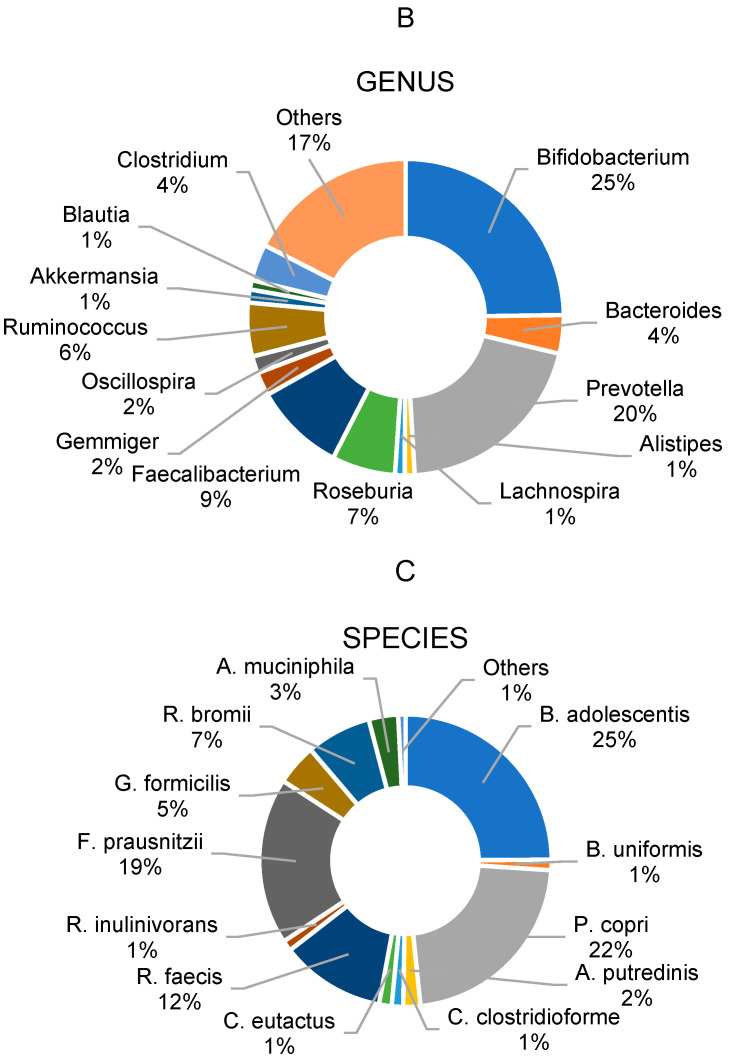
Gut microbiota composition of overweight and obese school children in study cohort at the phyla (**A**), genus (**B**), and species (**C**) levels. Only taxonomic groups above 1.0% are shown.

The most abundant bacterial phyla (Figure 2A) observed in the complete cohort of overweight and obese school children at baseline were, in order of highest relative abundances, *Firmicutes* (44.3%) and *Bacteroidetes* (26.9%), followed by *Actinobacteria* (25.6%) and *Proteobacteria* (1.1%). Concerning bacterial genera (Figure 2B), at baseline, the most abundant was *Bifidobacterium* (24.8%), followed by *Prevotella* (20.3%), *Faecalibacterium* (9.3%), *Roseburia* (6.5%) and *Ruminococcus* (5.5%). Regarding bacterial species (Figure 2C) at baseline, the most abundant was *Bifidobacterium adolescentis* (24.9%), followed by *Prevotella copri* (22.3%), *Faecalibacterium prausnitzii* (18.5%), *Roseburia faecis* (11.6%) and *Ruminococcus bromii* (7.2%).

### 3.3. Changes in Gut Microbiota Profile over the Intervention

#### 3.3.1. Overall Participants

**Figure 3:**
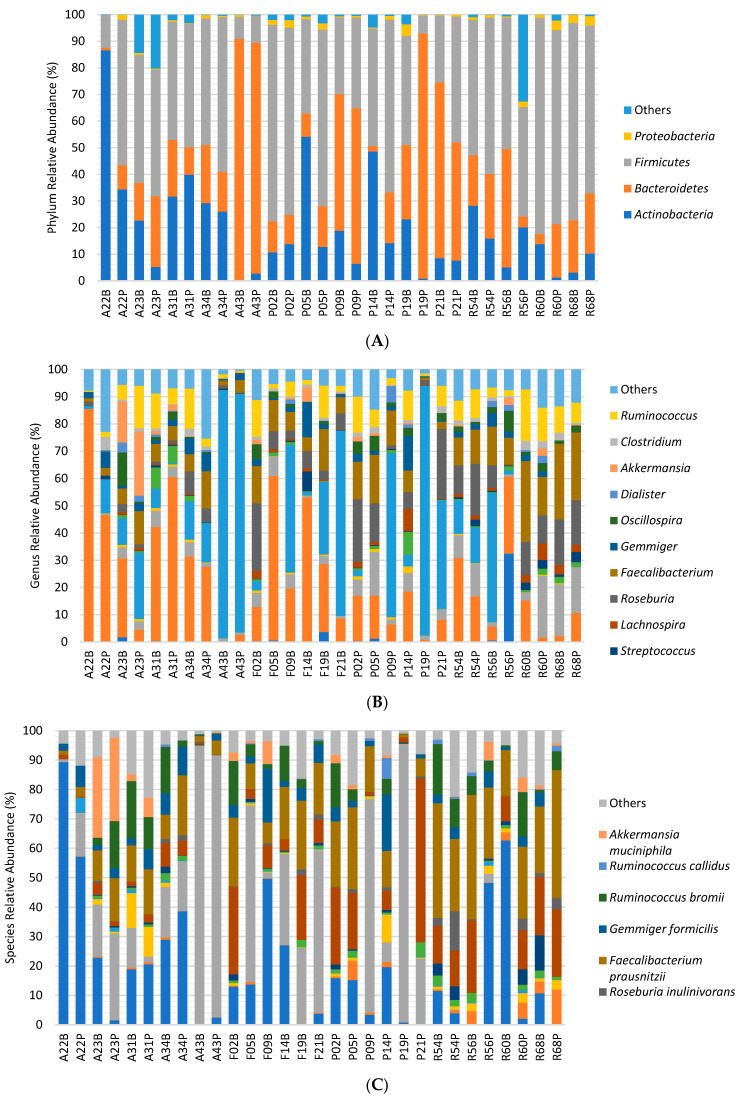
Phyla (**A**), genera (**B**) and species (**C**) relative abundance across all participants’ samples. Each sample is represented by one bar. Bars represent each taxa relative abundance. Each taxon is represented by a different colour. Baseline samples are identified with the letter B whereas samples from the post-intervention are identified with the letter P. All taxa with a relative abundance below 1% were grouped into Others.

The relative abundance of the most dominant bacterial phylum, genera and species per participant at baseline and end of the intervention is illustrated in Figure 3. Collectively (*n* = 15), from baseline to post intervention, no significant changes were found for any bacterial phylum (*p* > 0.05). The same was observed in the F/B ratio at the end of the intervention, which did not differ significantly from baseline (*p* > 0.05).

**Figure 4:**
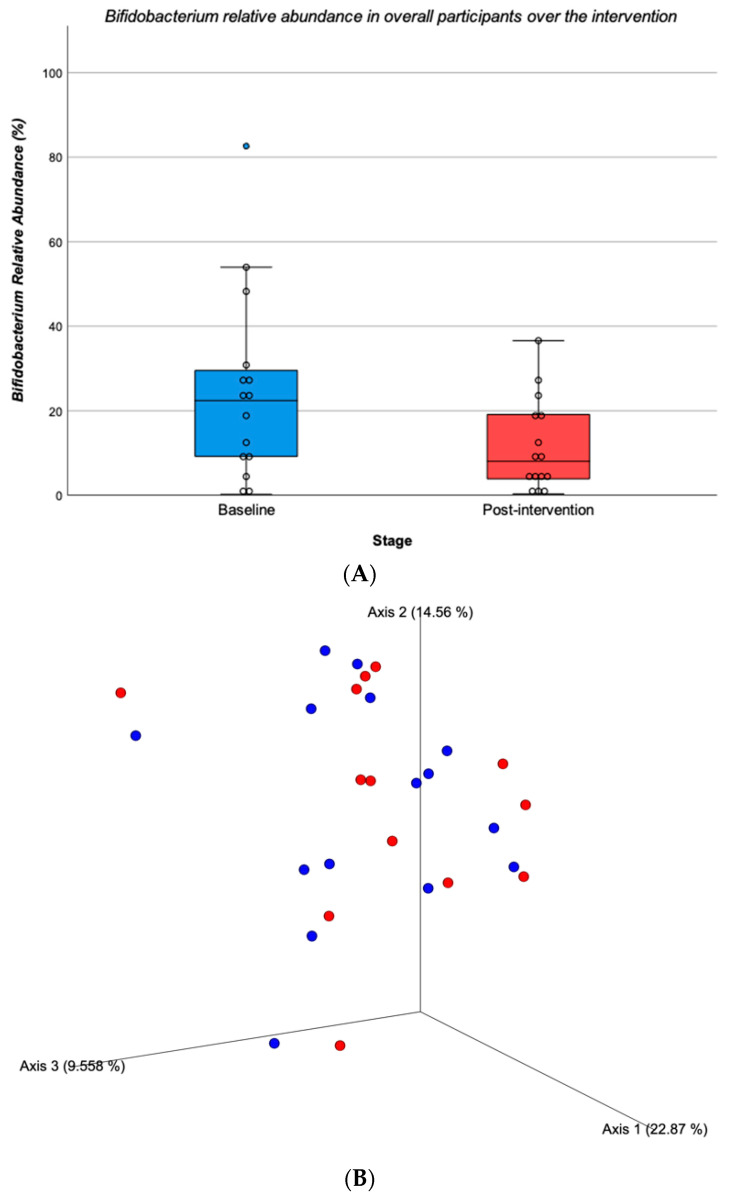
*Bifidobacterium* relative abundance in overall participants over the intervention (**A**); Principal coordinates analysis (PCoA) plot of the gut microbiota. Each point represents one sample. Baseline samples are coloured with blue dots and post-intervention samples are coloured with red dots. The percentage of the variance explained is indicated in parentheses in each axis (**B**).

Concerning bacterial genera, a significant decrease in *Bifidobacterium* was found for all participants over the intervention (Baseline = 24.780 ± 22.385 vs. Post = 11.949 ± 11.117; Z = −2.556, *p* = 0.011) (Figure 4A). Regarding bacterial species, no significant changes from baseline to the end of the intervention were found for any species (*p* > 0.05). The species richness at the end of the intervention did not differ significantly from baseline (Baseline = 62.467 ± 11.963 vs. Post = 61.933 ± 11.787; Z = −0.314, *p* = 0.753). Similarly, the alpha-diversity the Shannon index in the post-intervention did not differ significantly from baseline (Baseline = 4.478 ± 1.328 vs. Post = 4.574 ± 1.489; Z = −0.804, *p* = 0.422). A Principal Coordinate Analysis (PCoA) plot of the Bray–Curtis index distance was applied to obtain the principal coordinates and for the visualization of the complex relationships of gut microbiota composition at baseline and post-intervention (Figure 4B). The result suggests no differences between time points (*p* = 0.921).

#### 3.3.2. Football Group vs. Football and Nutrition Group

Table 2 shows the relative abundance of the topmost dominant bacteria between the baseline and post-intervention stages in the FG and NFG. No significant changes from baseline were found for any phylum in both groups (*p* > 0.05). Also, the F/B ratio at the end of the intervention did not differ significantly from baseline in any group (*p* > 0.05).

**Figure 5:**
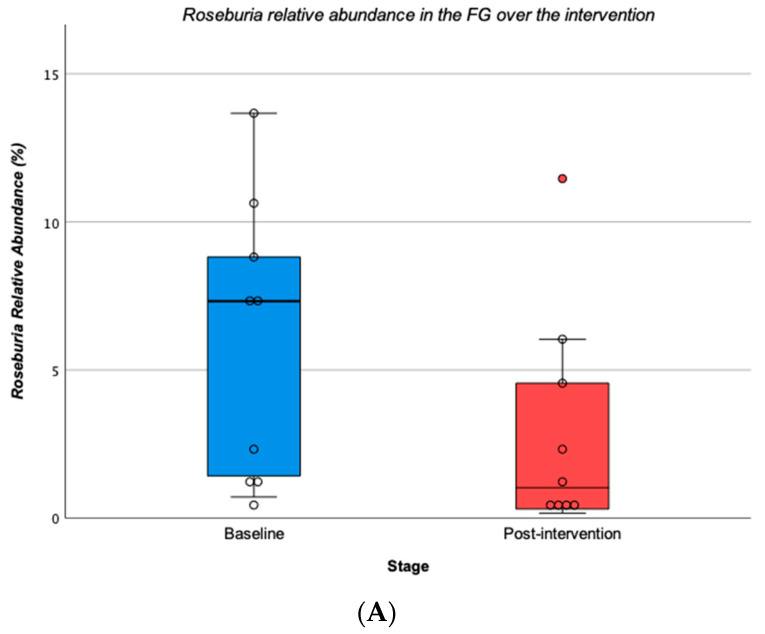

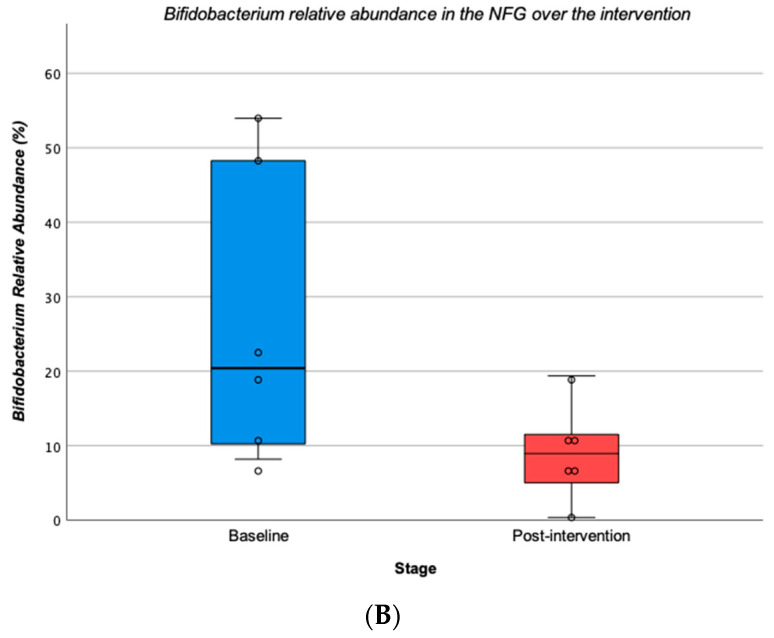
*Roseburia* relative abundance in the Football Group by stages (**A**) and *Bifidobacterium* relative abundance in the Nutrition and Football Group by stages (**B**).

Concerning bacterial genera, the FG significantly decrease *Roseburia* over the intervention (baseline = 5.919 ± 4.729 vs. post = 2.963 ± 3.820; Z = −2.310, *p* = 0.021) (Figure 5A), while the NFG, significantly decrease *Bifidobacterium* over the intervention (baseline = 26.918 ± 19.540 vs. post = 9.037 ± 6.421; Z = −2.201, *p* = 0.028) (Figure 5B). Regarding bacterial species, no significant changes from baseline to the end of the intervention were found for any species in both groups (*p* > 0.05). The species richness at the end of the intervention did not differ significantly from baseline analysing by groups (*p* > 0.05). Also, the Alpha-diversity Shannon index showed that alpha diversity at the end of the intervention did not differ significantly from baseline in both groups (*p* > 0.05).

### 3.4. Relationship between BMI Z-Score and the Gut Microbiota

**Figure 6:**
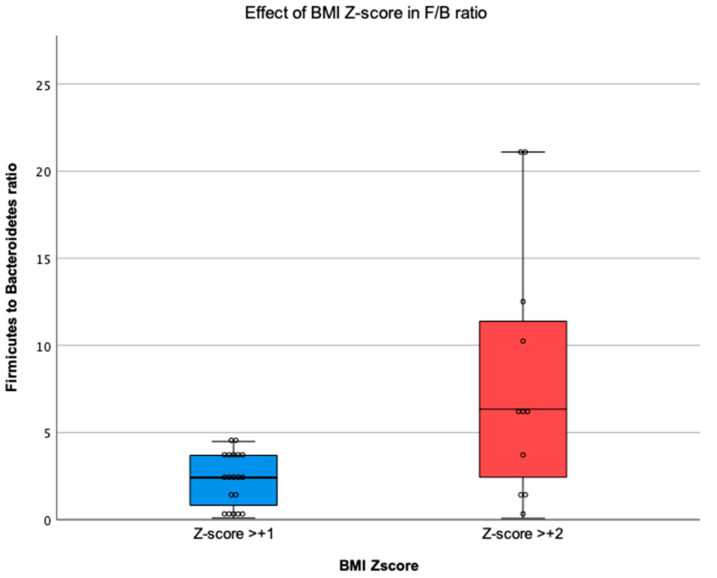
Boxplot of Firmicutes to Bacteroidetes ratio according to BMI Z-Score.

**Figure 7:**
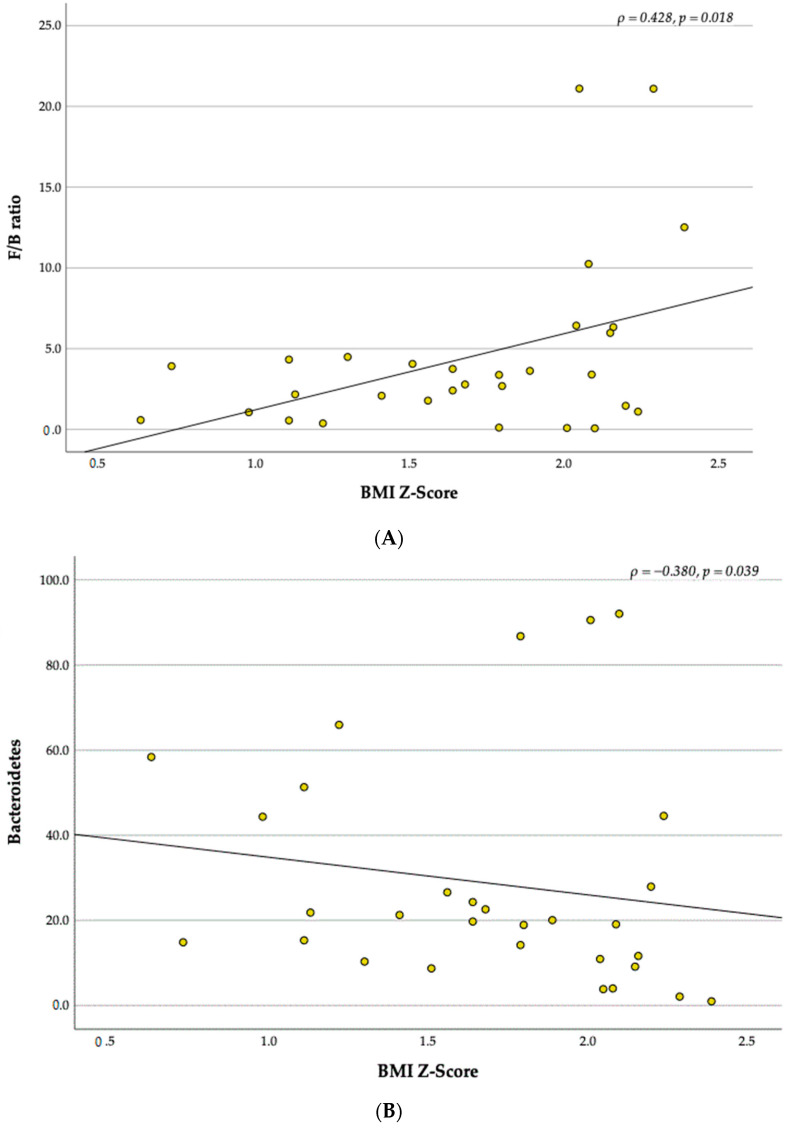
Correlations between the Firmicutes/Bacteroidetes ratio and BMI Z-Score (**A**). Correlations between the relative abundance of Bacteroidetes and BMI Z-Score (**B**).

Analysing children according to BMI Z-Score [32,33], we found that BMI Z-Score has a positive association in the F/B ratio (U = 51.00, *p* = 0.021). Children with a BMI Z-Score > +2 have a higher F/B ratio (8.16 ± 7.43) than children with a BMI Z-Score > +1 (2.33 ± 1.51) (Figure 6). Furthermore, a moderate positive correlation was found between BMI Z-Score and F/B ratio (ρ = 0.428, *p* = 0.018) (Figure 7A). Also, the relative abundance of Bacteroidetes was moderately negatively correlated with BMI Z-Score level (ρ = −0.380, *p* = 0.039) (Figure 7B).

### 3.5. Relationship between the MVPA and the Gut Microbiota

**Figure 8:**
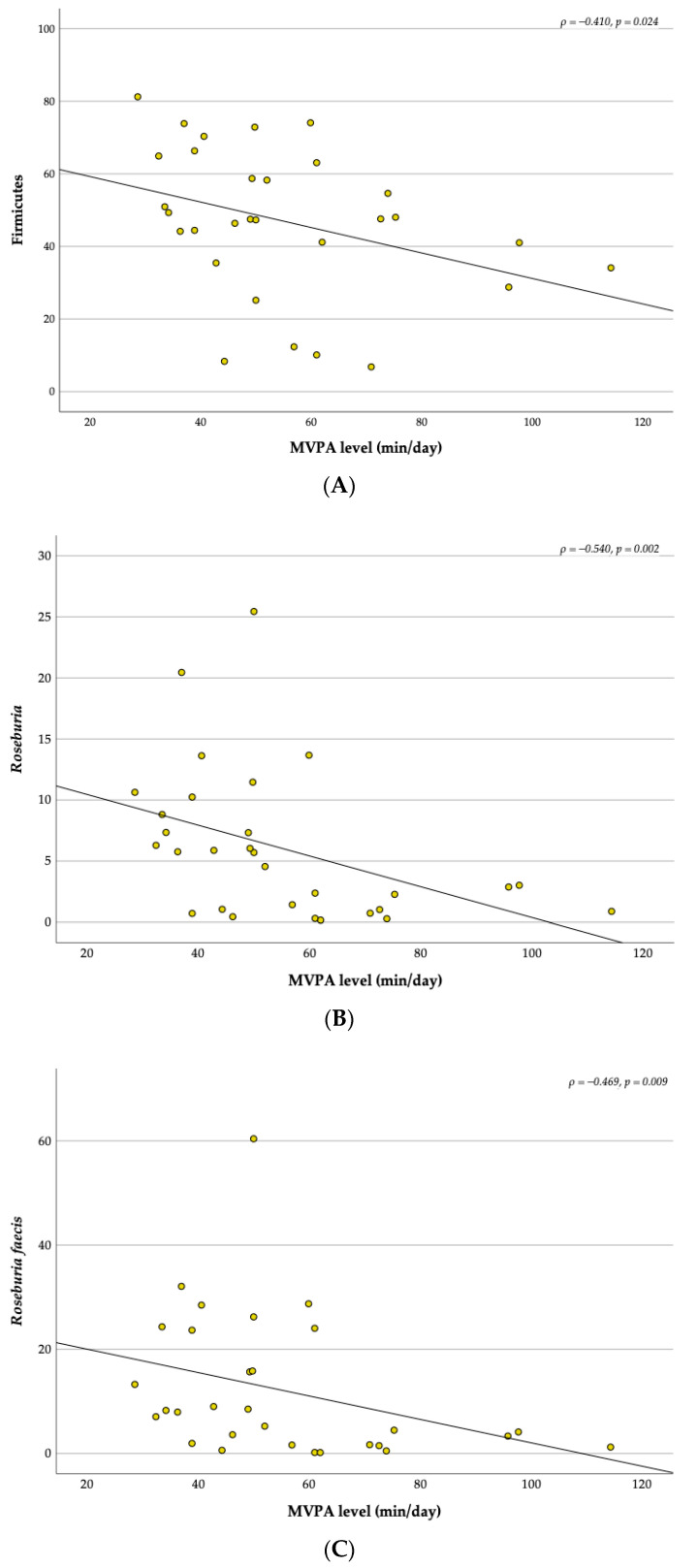

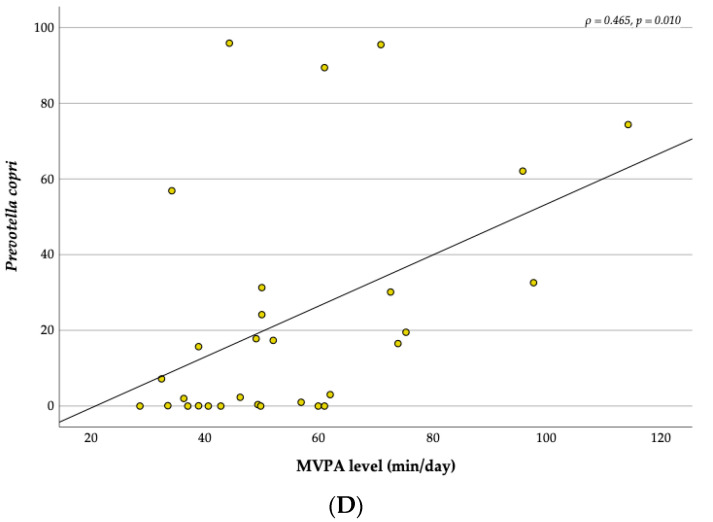
Correlations between the Firmicutes (**A**), *Roseburia* (**B**), *Roseburia faecis* (**C**) and *Prevotella copri* (**D**) with MVPA level.

Overall, the duration of each recreational football session was 60 min, two times per week, over 12 weeks. A relationship was found between the MVPA level and the relative abundance of the topmost abundant bacteria (Figure 8). MVPA level was moderately negatively correlated with the relative abundance of Firmicutes (ρ = −0.410, *p* = 0.024), *Roseburia* (ρ = −0.540, *p* = 0.002) and *Roseburia faecis* (ρ = −0.469, *p* = 0.009) and moderately positively correlated with *Prevotella copri* (ρ = 0.465, *p* = 0.010).

### 3.6. Relationship between the Dietary Intake and the Gut Microbiota

**Figure 9:**
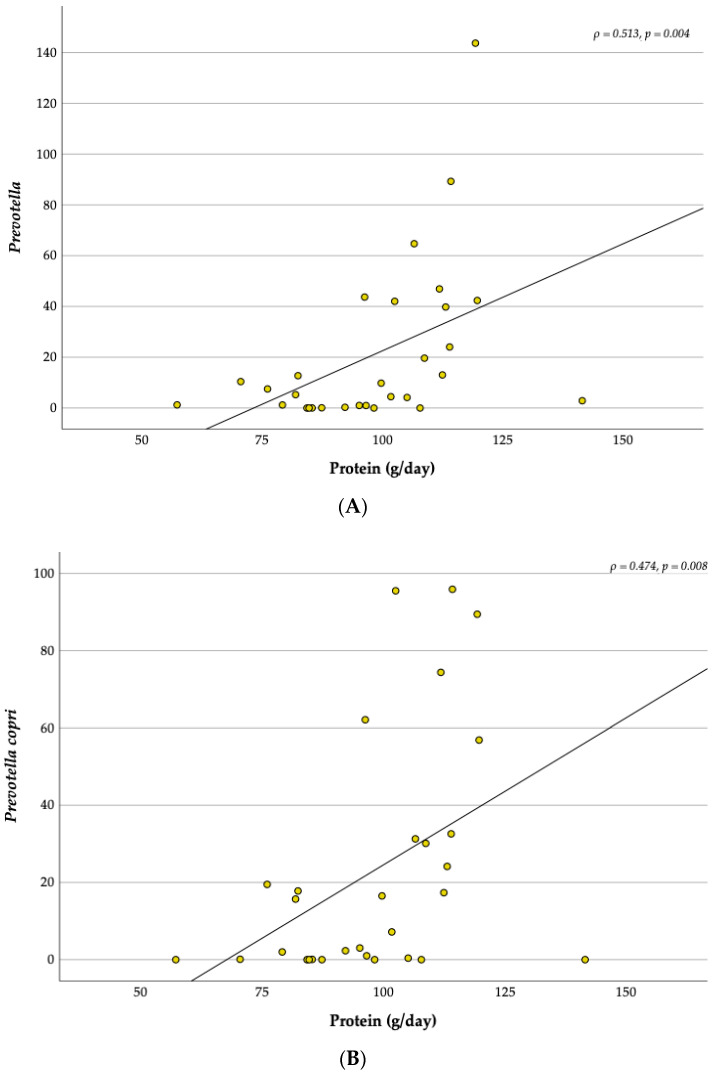
Correlations between Prevotella (**A**) and Prevotella copri (**B**) with protein intake.

The results of the 24 h recall are shown in Table 3. No significant differences at baseline and over the 12-weeks intervention were found between the FG and the NFG in the dietary intake (*p* > 0.05). However, it should be noted that fruit and vegetable consumption at baseline was close to presenting differences between groups (*p* = 0.077). Analysing the intervention in whole child population (*n* = 15), the results of the 24 h recall showed a significant decrease in carbohydrate intake (Z = −2.272, *p* = 0.023) and a significant increase in red meat consumption (Z = −2.106, *p* = 0.035) during the intervention. The relative abundance of *Prevotella* genus (ρ = 0.513, *p* = 0.004) and *Prevotella copri* spp. (ρ = 0.474, *p* = 0.008) was moderately positively correlated with the daily intake of protein (Figure 9). No significant correlations were found between food groups and the topmost relative abundant bacteria.

## 4. Discussion

To our knowledge, the current study was the first to characterize the gut microbiota of overweight and obese school children over a recreational football and nutritional education intervention program. Moreover, the current study showed that the multidisciplinary intervention promotes small but significant changes in the gut microbiota composition and in body composition of overweight and obese elementary school children.

### 4.1. Anthropometric and Body Composition

Over the intervention, both groups showed significant decreases in BMI, BMI z-score and WtHr. Similar lifestyle intervention studies with overweight and obese children with 10-week [51,52], 12-week [53], 6-month [21,54,55] found decreases in BMI, BMI z-score and/or WtHr. Additionally, both groups experienced reductions in %BFM, only the FG showed a significant decrease. Moreover, FG improves in FFM, MM and MVPA. This finding could be related to the fact that NFG started the intervention with a lower %BFM and a higher MM than FG. Improvements in body composition were observed in previous interventions, such as a 12-week recreational soccer program with obese adolescents where significant reductions in %BFM were discovered [53], and a 6-month intervention with obese boys where the active groups showed a significant decrease in %BFM and a significant increase in MM [54,55]. In addition to the improvements mentioned above, the NFG revealed significant differences in the reduction in waist circumference over the intervention, which has also been reported in other comparative investigations [53,54,55].

### 4.2. Gut Microbiota Composition in Overweight and Obese Children

In this study, the gut microbiota was mostly composed of bacteria from the Firmicutes phylum, followed by Bacteroidetes and Actinobacteria phylum. This composition is in accordance with other studies with obese children [18,56]. It has been suggested that an increase in F/B ratio can contribute to the pathophysiology of obesity [57]. Previous studies have shown a higher proportion of Firmicutes in obese children and lower proportion of Bacteroidetes, contributing to an elevated F/B ratio in obese children [12,56]. In our study the same was found at baseline. In contrast, other studies found no association [14].

The most abundant genera in our study at baseline were *Bifidobacterium*, *Prevotella, Faecalibacterium*, *Roseburia* and *Ruminococcus.* Likewise, in a study conducted by Riva et al. [56], *Bifidobacterium* and *Faecalibacterium* were also abundant, while Quiroga et al. [18] found Prevotella, Faecalibacterium and Ruminococcus between the most abundant genera in obese children.

*Bifidobacterium adolescentis* was the most prevalent species in our sample. In children aged 3 to 11 years, this species was more prevalent in obese children compared to lean ones [58]. Also, in a study with an obesity treatment program with overweight adolescents, *B. adolescentis* counts were significantly higher [51], which agrees with our findings. Another species that we found in high abundance was *Prevotella copri*. Interestingly, this species has rarely been reported in studies on overweight and obese children, although research on Chinese children has shown a higher relative abundance in the obese group compared to the normal weight group [59]. The third most abundant bacteria species in our sample was *Faecalibacterium prausnitzii*. Research shows contradictory results about this bacteria species in childhood obesity. This species has been found in greater relative abundance in overweight children [14,60] and was positively correlated with BMI z-score [56]. In contrast, other studies observed a higher relative abundance of *Faecalibacterium prausnitzii* in normal weight children than in obese children [61,62]. When comparing the gut microbiota between healthy children and healthy adults from the same region, the children’s gut microbiota was characterized by an enrichment in *Faecalibacterium prausnitzii* [63]. The fact that the literature shows contradictory results in obese samples may also be related to food intake. Obese children may have less or more abundance of *Prevotella* and *Faecalibacterium*, given that the major determinant for the abundance of these bacteria has been associated with food intake, such plant-based diets, rich in fibres [25,64,65,66]. Children who are obese may ingest an adequate amount of fibre and, therefore, promote the growth of these bacteria. In our study, particularly in the FG, fibre intake is higher than that recommended by the EFSA [67] for this age group (16 g).

The observed variations among studies could be attributed to differences in methodological approaches and experimental factors. For instance, small sample sizes and variability in the characteristics of study subjects, such as age, diet and geographic location, have been suggested as potential sources of variation [61,65,68]. Furthermore, differences in the primer sets and sequencing methods used or the existence of multiple phylotypes may also contribute to the discrepancies among findings [56,69,70].

### 4.3. Comparing the Gut Microbiota Composition between the Time Points

The decrease in the relative abundance of *Bifidobacterium* observed in the overall participants and in the NFG are in accordance with a study with adolescents who experienced a calorie-restricted diet (10–40%) and increased physical activity (15–23 kcal/kg body weight/week) over 10 weeks, that led to decreased *Bifidobacterium* species counts, namely, *Bifidobacterium longum* and *Bifidobacterium adolescentis* [51]. Previous studies described a relation between the reductions in *Bifidobacteria* and the decrease in carbohydrates intake [71]. In fact, the whole child population of our study significantly decreased the carbohydrate intake over the intervention, which could partially explain the reductions in the relative abundance of Bifidobacterium by the consequence of insufficient substrate to support growth [71], which was also observed in an intervention study that combined physical activity and nutritional education or reduced caloric intake [21,51].

Concerning the significant decrease in *Roseburia* in the FG over the intervention, upon analysing possible relationships, we verified that MVPA level was negatively associated with the relative abundance of Firmicutes phylum, *Roseburia genus* and *Roseburia faecis species*. In our study with overweight and obese children, the program contributed to the decrease in this bacterium in the FG. In a 12-week (two sessions per week) program of strength and endurance combined training, an increase in the Firmicutes phylum was observed [18], and the combination of physical activity and nutritional counselling result in fat loss and increase the proportions of Firmicutes phylum [20]. Although this finding contrasts with other studies, as reported by Quiroga et al. [18]. In their study, the training program increased some genera, such as *Roseburia* [18]. It should be noted that this finding was observed in the FG, which was also the group that significantly increased the MVPA level throughout the intervention. On the other hand, the relative abundance of *Roseburia* spp., a bacterium with a dominant role in butyrate production, decreases as carbohydrate intake decrease [71], which could also be related with the significant decrease in carbohydrate intake observed in the present study.

The species richness, alpha and beta diversity did not differ from baseline to the end of the 12-week intervention (*p* > 0.05), in accordance with a 2-month weight reduction program with obese children [20]. However, while some authors report that overweight and obese children tend to have a lower bacterial diversity [11,59,61], others have not found significant differences between obese and normal-weight children [14,21,56,72]. Regarding alpha-diversity Shannon index, we observed that our sample presented a higher diversity than other studies with overweight and obese children [56,72].

### 4.4. Firmicutes to Bacteroidetes Ratio and BMI Z-Score

The difference in F/B ratio over the intervention was not significant in our study. However, there was a reduction in this ratio which could be partially attributed to the reduction in BMI Z-score, as verified by the positive correlation between BMI Z-score groups and the F/B ratio, showing that children with a higher BMI Z-score level have a higher F/B ratio, whereas children with lower BMI Z-score level have a lower F/B ratio. Previous studies found the same relation regarding BMI when compared normal-weight and obese children [12,56]. Although the F/B ratio was higher in children with a higher BMI Z-score, we found a considerable variation in the ratio, which had also been reported by Riva et al. [56]. This variation suggest that the F/B ratio may not be a solid marker for obesity [56]. Moreover, we found a negative correlation between the BMI Z-score level and the relative abundance of Bacteroidetes that matches the findings in the study conducted by Riva et al., who report that BMI z-score was negatively correlated with Bacteroidetes [56]. This finding may explain why the increased F/B ratio is found in children with higher BMI Z-scores.

### 4.5. Correlations with Physical Activity and Diet

The MVPA level was positively correlated with *Prevotella copri*, a bacterium species belonging to *Prevotella genus* and Bacteroidetes phylum. As previously mentioned, obese children seem to have a lower proportion of Bacteroidetes [12,56], so we hypothesize that physical activity tends to modify the relative abundance of Bacteroidetes phylum. In previous studies, the *Bacteroides-Prevotella* group was observed to increase after multidisciplinary obesity treatment programme in the adolescents who lost more than 4 kg [52].

Concerning diet, *Prevotella genus and Prevotella copri* spp. also showed a positive correlation with the daily intake of protein. To our knowledge, this correlation is a novelty in research with overweight and obese children after a multidisciplinary program. Additionally, none of the previous lifestyle interventions with obese children reported this relationship between protein intake and *Prevotella copri*; it has been suggested that increased *Prevotella* abundance was positively correlated with several amino acid metabolism pathways, including branched-chain amino acid metabolism [73].

There are conflicting reports that implicate *Prevotella genus* and *Prevotella copri*. This bacterium is a common human gut microbe that has been both positively and negatively associated with host health [74,75]. Recently, *Prevotella copri* has been demonstrated to encompasses at least four distinct species-level lineages and exhibits a high diversity [74]. The relationship between *Prevotella copri*, physical activity and diet is not yet fully understood, and more research is needed to clarify this relationship.

There are several factors that may have contributed to the results observed in our study. Firstly, although the level of physical activity between the groups was not significantly different at baseline, it appeared that the NFG was more active than the FG. Secondly, even though nutritional education did not directly intervene in dietary intake and only one of the groups received these sessions, there seemed to be greater attention paid to food intake by all the participants. Lastly, the small sample size and duration of the intervention may have limited the potential impact of the study’s findings.

## 5. Conclusions

Our findings suggest that a 12-week combined intervention with physical activity and nutritional education improved body composition parameters and is capable of inducing significant and positive changes in the composition of gut microbiota in overweight and obese school children. Childhood seems to represent a transitional period for the gut microbiota, and it is important to provide conditions and lifestyles for the development of a healthy microbiota, highlighting the importance of physical activity and lifestyle interventions as a non-pharmacological therapy in childhood obesity.

## Figures and Tables

**Table 1:** Comparison of the results of anthropometric, body composition, and physical activity measurements between the pre- and post-intervention stages in the FG and NFG.

	FG (*n* = 9)					NFG (*n* = 6)				
Variables	Baseline *		Post *		%Δ	Baseline *		Post *		%Δ
Age (y)	8.8	±0.8	9.2	±0.8		9.4	±0.2	9.7	±0.2	
Height (cm)	134.8	±11.2	138.1	±11.2	2.4	141.9	±1.4	144.5	±2.0	1.8
Weight (kg)	41.9	±11.9	42.7	±11.8	1.8	46.8	±6.6	46.6	±7.0	−0.6
BMI (kg/m^2^)	22.6	±2.9	22.0	±2.7 †	−2.8	23.3	±3.3	22.3	±3.4 †	−4.1
BMI Z-score	1.8	±0.4	1.6	±0.4 †	−10.0	1.7	±0.5	1.5	±0.7 †	−14.7
WC (cm)	76.1	±11.2	75.7	±10.8	−0.5	78.8	±7.1	74.5	±9.0 †	−5.4
WtHr	0.6	±0.0	0.5	±0.0 †	−2.8	0.6	±0.0	0.5	±0.1 †	−7.0
%BFM	34.8	±4.8	31.8	±5.7 †	−8.6	30.5	±6.2	29.7	±7.3	−2.5
FFM (kg)	27.6	±9.1	29.4	±9.5 †	6.6	32.3	±2.8	32.3	±2.4	0.1
MM (kg)	17.9	±6.1	19.1	±6.3 †	6.3	21.9	±2.0	21.2	±2.1	−3.3
MVPA (min/d)	46.7	±15.1	58.6	±10.1 †	25.5	59.9	±28.9	57.9	±30.7	−3.5

* Data are presented as mean ± standard deviations. Abbreviations: FG, football group; NFG, nutrition and football group; Post, post intervention; BMI, Body mass index; WC, waist circumference; WtHr, Waist-to-height ratio; %BFM, body fat mass percentage; FFM, fat-free mass; MM, muscle mass; MVPA, moderate to vigorous physical activity. † Significant within-group difference.

**Table 2:** Relative abundance of the topmost dominant bacteria between the baseline and post-intervention stages in the FG and NFG.

	FG (*n* = 9)					NFG (*n* = 6)				
	Baseline		Post		%Δ	Baseline		Post		%Δ
phylum level										
Actinobacteria	24.5	±26.2	17.3	±13.9	−29.5	27.3	±19.4	9.3	±5.3	−66.1
Bacteroidetes	26.2	±27.1	24.3	±24.6	−7.3	28.0	±25.7	40.0	±31.5	43.2
Firmicutes	46.3	±24.0	50.3	±17.8	8.8	41.4	±17.4	48.3	±24.5	16.6
Proteobacteria	1.0	±0.9	1.5	±1.4	47.7	1.4	±1.6	1.3	±1.0	−1.7
F/B ratio	5.4	±6.9	3.9	±2.9	−27.7	5.7	±7.9	2.6	±2.5	−53.1
genus level										
Bifidobacterium	23.4	±25.2	13.9	±13.4	−40.5	26.9	±19.5	9.0	±6.4 †	−66.4
Bacteroides	4.4	±4.7	4.3	±7.9	−3.0	3.2	±2.3	4.7	±4.1	44.5
Prevotella	18.7	±29.5	21.3	±46.4	13.7	22.8	±26.7	22.4	±22.6	−1.6
Roseburia	5.9	±4.7	3.0	±3.8 †	−49.9	7.3	±6.6	9.5	±9.3	31.0
Faecalibacterium	9.7	±8.9	7.1	±4.7	−27.3	8.6	±3.6	7.1	±4.7	−17.5
Ruminococcus	6.0	±5.4	3.8	±4.9	−36.7	4.7	±4.4	4.7	±3.5	0.1
species level										
Bifidobacterium adolescentis	22.2	±28.8	21.5	±24.8	−3.4	29.0	±25.1	10.3	±9.9	−64.4
Prevotella copri	23.0	±32.8	17.7	±28.9	−23.1	21.3	±25.2	33.6	±41.3	57.3
Roseburia faecis	10.2	±10.2	7.4	±8.8	−27.2	13.8	±12.2	20.4	±22.7	48.0
Faecalibacterium prausnitzii	19.2	±16.7	21.7	±13.2	12.8	17.5	±8.3	15.9	±12.0	−8.9
Ruminococcus bromii	7.8	±8.9	8.2	±7.1	5.1	6.5	±6.4	4.6	±6.3	−29.4

Data are presented as mean ± standard deviations. Abbreviations: FG, football group; NFG, nutrition and football group; Post, post-intervention; F/B ratio, Firmicutes/Bacteroidetes ratio. † Significant within-group difference.

**Table 3:** Daily energy and nutrient intake between the baseline and post-intervention stages in the FG and NFG.

	FG (*n* = 9)					NFG (*n* = 6)				
	Baseline		Post		%Δ	Baseline		Post		%Δ
Macronutrients										
Energy (kcal)	1916.2	±362.9	1816.0	±379.0	−5.2	1909.1	±285.8	1704.3	±329.1	−10.7
Protein (g)	92.6	±17.6	101.7	±10.8	9.7	104.1	±22.2	95.3	±21.1	−8.5
* Carbohydrate (g)	259.7	±78.1	203.1	±61.6	−21.8	247.9	±68.4	203.6	±46.3	−17.9
Fiber (g)	17.3	±9.2	14.8	±7.3	−14.5	14.6	±5.7	13.5	±5.2	−7.2
Sugar (g)	89.9	±57.4	67.3	±36.7	−25.1	81.7	±31.7	61.8	±17.2	−24.4
Fat (g)	56.9	±14.5	66.7	±17.2	17.1	56.6	±13.4	55.5	±16.0	−2.0
Saturated Fat (g)	18.9	±4.6	21.7	±4.6	14.6	18.5	±6.9	19.9	±6.1	7.1
Food Groups										
Fruits and Vegetables (g)	653.9	±238.6	516.7	±430.4	−20.9	364.2	±329.5	374.0	±262.2	2.7
* Red Meat (g)	72.8	±87.1	134.8	±94.9	85.2	60.7	±66.9	155.0	±90.7	155.4
Cheese and Yoghurt (g)	122.9	±123.8	140.1	±115.6	14.0	109.0	±115.5	144.8	±128.9	32.8
Dairy (g)	361.3	±103.6	408.4	±149.6	13.0	453.0	±269.9	476.3	±140.3	5.1

Data are presented as mean (±SD). FG, football group; NFG, nutrition and football group; Post, post-intervention. * Significant difference in whole population over the intervention (*p* < 0.05).

## Data Availability

The original contributions presented in the study are included in the article. Further inquiries can be directed to the corresponding authors.
